# Increased Levels of IL-16 in the Central Nervous System during Neuroinflammation Are Associated with Infiltrating Immune Cells and Resident Glial Cells

**DOI:** 10.3390/biology10060472

**Published:** 2021-05-27

**Authors:** Shehla U Hridi, Mark Barbour, Chelsey Wilson, Aimee JPM Franssen, Tanith Harte, Trevor J Bushell, Hui-Rong Jiang

**Affiliations:** Strathclyde Institute of Pharmacy and Biomedical Sciences, University of Strathclyde, 161 Cathedral Street, Glasgow G4 0RE, UK; shehla.hridi@gmail.com (S.U.H.); markbarbour9488@gmail.com (M.B.); chelsey.wilson@strath.ac.uk (C.W.); aimeefranssen@gmail.com (A.J.F.); tanith.harte@strath.ac.uk (T.H.); trevor.bushell@strath.ac.uk (T.J.B.)

**Keywords:** IL-16, experimental autoimmune encephalomyelitis, central nervous system, immune cells, glial cells

## Abstract

**Simple Summary:**

Interleukin-16 is a protein involved in the migration of some immune cells and plays an important role in the development of multiple sclerosis, an inflammatory demyelinating disease that affects the central nervous system (i.e., brain and spinal cord). Currently, it is not fully understood which cells produce interleukin-16 during the inflammatory response in the central nervous system. This study investigates the correlation between the expression levels of interleukin-16 and the severity of neuroinflammation and determines the cells which produce interleukin-16, using a mouse model of multiple sclerosis. Our data show that the expression levels of interleukin-16 are significantly increased in the brain and spinal cord tissues of the mouse model compared to controls. Furthermore, other immune assays reveal that the significantly increased number of cells expressing interleukin-16 in the central nervous system lesions are likely to be infiltrating immune cells and resident glial cells, but not neurons. Our findings suggest that interleukin-16 is closely involved in the pathology of multiple sclerosis and other inflammatory diseases in the central nervous system via the glial and infiltrating immune cells.

**Abstract:**

Interleukin (IL)-16, a CD4^+^ immune cell specific chemoattractant cytokine, has been shown to be involved in the development of multiple sclerosis, an inflammatory demyelinating disease of the central nervous system (CNS). While immune cells such as T cells and macrophages are reported to be the producers of IL-16, the cellular source of IL-16 in the CNS is less clear. This study investigates the correlation of IL-16 expression levels in the CNS with the severity of neuroinflammation and determines the phenotype of cells which produce IL-16 in the CNS of experimental autoimmune encephalomyelitis (EAE) mice. Our data show that IL-16 expression is significantly increased in the brain and spinal cord tissues of EAE mice compared to phosphate buffered saline (PBS) immunised controls. Dual immunofluorescence staining reveals that the significantly increased IL-16^+^ cells in the CNS lesions of EAE mice are likely to be the CD45^+^ infiltrating immune cells such as CD4^+^ or F4/80^+^ cells and the CNS resident CD11b^+^ microglia and GFAP^+^ astrocytes, but not NeuN+ neurons. Our data suggest cytokine IL-16 is closely involved in EAE pathology as evidenced by its increased expression in the glial and infiltrating immune cells, which impacts the recruitment and activation of CD4^+^ immune cells in the neuroinflammation.

## 1. Introduction

Multiple sclerosis (MS) is an autoimmune, demyelinating disease which affects more than 2.2 million people worldwide [1]. Experimental autoimmune encephalomyelitis (EAE) is the most commonly used animal model for studying MS as many studies have suggested its close immunopathological resemblance to that found in MS [2,3]. In MS/EAE, the inflammatory response is primarily driven by myelin specific T cells and B cells infiltrating the central nervous system (CNS) by crossing the blood brain barrier [4]. Following entry, the lymphocytes are reactivated by antigen-presenting cells in the CNS to release pro-inflammatory mediators, which induce activation of resident microglia and astrocytes as well as other infiltrating immune cells. This cascade of inflammatory response often leads to myelin and axonal damage [5,6,7].

Interleukin (IL)-16 is a cytokine that was initially identified as a lymphocyte chemoattractant factor for T cells [8,9]. IL-16 is predominantly produced by CD4^+^ and CD8^+^ cells [10] as a precursor protein known as pro-IL-16, which is enzymatically cleaved by caspase 3, releasing the bioactive mature form of IL-16 [11]. Other cells that can produce IL-16 include monocytes/macrophages, dendritic cells (DCs), mast cells, fibroblasts and microglia [12]. IL-16 is a ligand for the CD4 receptor, and cell surface expression of CD4 is required to exert the bioactivities of IL-16 observed in immune cells [12]. Through interaction with CD4, IL-16 can act as a chemoattract to a variety of CD4^+^ immune cells. IL-16 may also function as a ligand of CD9 as reported in human lung cancer cells [13].

IL-16 has been designated as a pro-inflammatory cytokine that activates immune T cells [9] and macrophages [14]. Several studies have suggested that IL-16 is involved in the development of various autoimmune diseases including MS [12,15,16]. IL-16 is found to be secreted from MS patient-derived cytotoxic T lymphocytes (CTLs) when stimulated with the myelin-specific antigen proteolipid protein, suggesting a role for IL-16 in MS disease [17]. Furthermore, anti-IL-16 antibody treatment has been shown to ameliorate relapses in EAE mice and diminish the CNS infiltration by CD4^+^ T cells [18]. However, conditioned media from lymphocyte preparations containing IL-16 are shown to be neuroprotective against kainate-induced excitotoxicity and neuronal death [19]. In addition, recombinant IL-16 reduces neuronal excitability and synaptic activity in mice primary hippocampal cultures [20]. This study examines whether the expression of IL-16 is altered in the brain and spinal cord of EAE mice. We also determine whether IL-16 expression correlates with neuroinflammation and identify the cellular sources of IL-16 within the CNS. Findings from this study suggest the close involvement of IL-16 in EAE development, which is likely to contribute to CNS neuroinflammation via both the infiltrating immune and the CNS resident cells.

## 2. Materials and Methods

### 2.1. Mice and EAE Induction

Naïve C57BL/6J mice were bred and maintained in the Biological Procedure Unit at the University of Strathclyde. Female animals at eight weeks of age were used in all experiments. All animal experiments were performed under the guidelines of the UK Animals (Scientific Procedures) Act 1986 and were conducted under a Project License granted by the UK Home Office and with ethical approval from the University of Strathclyde Animal Welfare Ethical Review Body.

EAE was induced as previously described [21]. Mice were immunized s.c. on day 0 with 100 µL of 100 µg MOG_35-55_ (ChinaPeptides Co Ltd., China) emulsified in complete Freunds adjuvant (CFA; Merck, Germany) supplemented with 500 µg *mycobacterium tuberculosis* (BD Biosciences, UK). In addition, each mouse received 100 ng pertussis toxin (PTX; Tocris Bioscience, UK) in 100 µL PBS injected i.p. on day 0 and again on day 2. PBS control mice received 100 µL of PBS emulsified in the above CFA without MOG_35-55_, together with the two doses of PTX injection. The number of animals used in each experiment varied from 3 to 15 and is detailed in each figure legend. Mice were monitored daily for signs of disease development, and clinical scores were recorded based on the following evaluation system: 0 = no clinical sign; 0.5 = partial loss of tail tone; 1.0 = complete loss of tail tone; 1.5 = altered gait; 2.0 = hind limb weakness; 2.5 = paralysis of one leg; 3.0 = hind limb paralysis; 3.5 = hind limb paralysis with significantly reduced mobility; 4.0 = forelimb involvement; 5.0 = moribund. There was no unexpected mortality in our studies.

### 2.2. Brain and Spinal Cord Tissue Preparation for Immunohistochemistry

PBS or MOG_35-55_ immunised mice were sacrificed via asphyxiation in a CO_2_ chamber on day 12 (EAE onset), 16 (EAE peak) and 26 (EAE resolution) post immunisation. Animals were first perfused with PBS, after which brains were harvested and spinal cords flushed out with PBS by hydrostatic pressure using a sterile 19G needle. After collection, tissues were placed in Optimal Cutting Temperature compound (OCT) mounting medium and frozen on dry ice before being cut into 7-µM-thick sections on a Shandon cryotome (Thermo Fisher Scientific, UK).

### 2.3. Immunohistochemistry

Frozen tissue sections were stained with antibodies against CD4 (eBioscience, UK, # 14-0042-85), CD45 (eBioscience, UK, # 14-0451-85) and IL-16 (Thermo Fisher Scientific, UK, # PA5-20670, detecting both pro-IL-16 and bioactive IL-16) followed by incubation with an appropriate biotin-conjugated secondary antibody (eBioscience, UK), horseradish peroxidase and ImmPACT AMEC red peroxidase substrate (Vector Laboratories, USA) for detection. Finally, sections were counterstained in Gill 2 haematoxylin (Merck, Germany). The stained slides were viewed using a Nikon Eclipse 50 bright field microscope. Images of three different magnifications (×10, ×20, and ×40) were taken using a digital camera (Nikon Digital Sight DS-U3) connected to a computer containing NIS Element F 3.2 imaging software.

For immunofluorescence staining, frozen tissue sections were stained with antibodies against CD4 (Thermo Fisher Scientific, # 14-0451-85), CD11b (Thermo Fisher Scientific, # 14-0112-85), CD45 (Thermo Fisher Scientific, # 14-0451-85), F4/80 (Thermo Fisher Scientific, # 14-4801-85), GFAP (Cell Signalling Technology, UK, # 36708), NeuN (Merck Millipore, Germany, # MAB377) and IL-16 (Thermo Fisher Scientific, # PA5-20670). This was followed by incubation with appropriate Alexa Fluor-conjugated secondary antibodies (Thermo Fisher Scientific). Finally, sections were mounted with vectashield containing DAPI (Vector Laboratories). Slides were then visualized on a Nikon Eclipse E600 epiflorescent microscope.

### 2.4. Quantification of Positive Staining Cells in the CNS

All quantification of positive staining was carried out using ImageJ. Regions of interest (ROIs) were determined by using fixed size and location were evenly distributed across the relevant areas depending on the tissue and particular area being analysed. For the spinal cord, ten ROIs were assigned across the white and grey matter of each spinal cord. For cerebellum analysis, three ROIs were used in each of the cerebellar white matter, granular layer and molecular layer per mouse. Finally, three ROIs were used for each of hippocampal C1, C2 and C3 regions and two areas of dentate gyrus for each mouse. ROIs were comparable between individual mice.

The total number of cells and the number of either single or double positive cells within each ROI were counted and recorded. The percentage of positive cells within each tissue section was presented in the figures, with n representing the number of animals.

### 2.5. Tissue Preparation for ELISA

Spleens were harvested from EAE mice and PBS controls on day 16 and disrupted to form a single cell suspension. Splenocytes were cultured in 24-well plates at 2 × 10^6^ cells/well with media alone, media with 50 μg/mL MOG_35-55_ or media with 50 µg/mL Concanavalin A (ConA) (SigmaAldrich, UK) and incubated at 37 °C in a cell incubator with 5% CO_2_. After 72 h, cell supernatants were collected.

Spinal cords and brain (divided into cerebellum and cerebrum) tissues were collected from EAE mice and PBS control mice on day 16 and then homogenised in PBS containing 10 µL/mL Halt™ Protease and Phosphatase Inhibitor Cocktail (Thermo Fisher Scientific) and centrifuged at 13,000 rpm for 15 min. Supernatants were then collected.

The levels of IL-16 in the supernatants collected from cell culture and CNS tissues were detected by ELISA following the manufacturers’ guidelines (R&D Systems, Mouse IL-16 Duoset ELISA, # DY1727, calibrated against a sequence of bioactive IL-16, which is also part of the pro-IL-16.)

### 2.6. Statistics

All statistics were carried out using Graph Pad Prism software. All data are expressed as mean ± S.E.M. in all experiments. EAE clinical data were analysed using two-way repeated measures ANOVA with Bonferroni post-hoc test where required. Statistical analysis of all other data was performed using one-way ANOVA with Bonferroni post-hoc where required, with *p* < 0.05 being taken as indicative of statistical significance.

## 3. Results

### 3.1. The Levels of IL-16 in CNS Tissues of EAE Mice Are Elevated and Correlate with the Severity of CNS Inflammation

**Figure 1:**
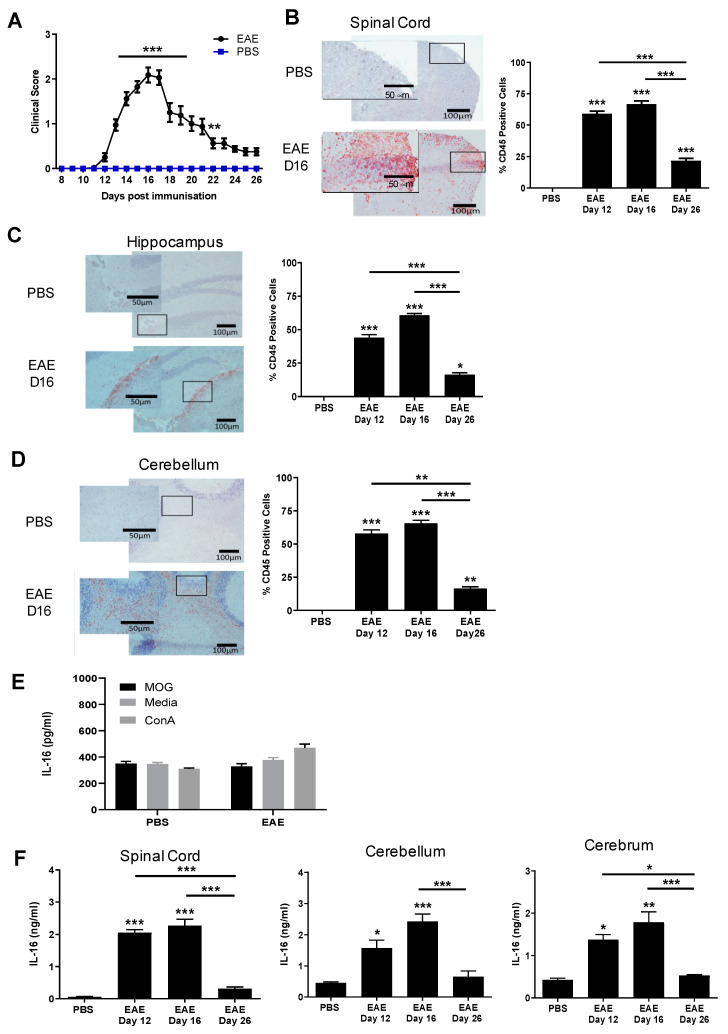
Induction of EAE and IL-16 production by tissues of EAE mice. (**A**) C57BL/6J female mice were injected with MOG_35-55_ emulsified in CFA, together with PTX. Clinical symptoms were recorded over the course of 26 days. Control mice received PBS + CFA + PTX immunisation. N = 15. Statistical significance was determined by one-way ANOVA with repeated measures and Bonferroni post-hoc tests. ** *p* < 0.01, *** *p* < 0.001. (**B**–**D**) CNS tissues were harvested from PBS control mice on day 16 and EAE mice on day 12, day 16 and day 26. Spinal cord (**B**), hippocampus (**C**) and cerebellum (**D**) tissues were stained for CD45 and counterstained with haematoxylin. Representative images are shown. The percentage of CD45^+^ cells was quantified for each group; results are expressed as mean ± SEM. N = 3 for all groups. Statistical significance was determined by one-way ANOVA with Bonferroni post-hoc tests. (**E**) Spleen cell suspensions prepared from day 16 of PBS or MOG_35-55_ immunised mice were incubated either with media alone or media containing MOG_35-55_ (50 μg/mL), or ConA (50 μg/mL). Supernatants were collected after 72 h of culture and used to detect IL-16 production by ELISA. Results are expressed as the mean ± SEM. PBS n = 4 and EAE n = 6. Statistical significance was determined by one-way ANOVA with Bonferroni post hoc tests. (**F**) Supernatants from homogenised spinal cord, cerebellum or cerebrum were collected and prepared to analyse IL-16 levels by ELISA. PBS n = 3, EAE n = 5 at each time point. Statistical significance was determined by one-way ANOVA with Bonferroni post hoc tests. * *p* < 0.05, ** *p* < 0.01, *** *p* < 0.001 versus PBS or comparison groups are indicated by the bar lines.

Mice immunised with PBS emulsified in CFA together with the injection of PTX did not develop EAE clinical signs while mice immunized with MOG_35-55_ developed a monophasic disease course with EAE onset occurring around day 12 (onset stage) with a disease incidence rate of 100% by day 15 post immunisation (Figure 1A). Disease severity reached its peak on day 16 (peak stage), after which the mice began to gradually recover, with a steady decrease in the clinical score (day 26, resolution stage). Histological analysis with anti-CD45 staining confirmed the extent of inflammation and the levels of cellular infiltration in the spinal cord (Figure 1B), brain hippocampus and cerebellum tissues (Figure 1C,D) over the course of EAE, particularly at the peak stage, while very few CD45^+^ cells or inflammation were observed in the CNS of control group mice.

Immune cells of EAE mice produce increased antigen-specific cytokines following *in vitro* stimulation [22], and we also observed antigen-specific increases in IFN-γ, IL-17 and IL-6 production following MOG_35-55_ peptide stimulation by EAE splenocytes compared to PBS and naive controls (data not shown). However, we sought to focus on investigating the production of IL-16 using an ELISA kit from R&D Systems detecting both pro-IL-16 and bioactive IL-16. Spleen cells from both PBS and EAE mice groups on day 16 post immunisation demonstrated consistently high levels of IL-16 production with no statistical difference between the EAE and PBS control groups with or without MOG_35-55_ in the culture (Figure 1E). Cells stimulated with ConA in the culture induced similar levels of IL-16 production when compared with cells treated with MOG_35-55_ or medium alone. The data suggest that IL-16 is constitutively released by splenocytes in PBS or MOG peptide immunised mice.

We then examined the levels of IL-16 in spinal cord, cerebellum and cerebrum of EAE on day 12, day 16 and day 26 of disease onset, peak and resolution stages respectively, together with tissues of PBS-immunised control mice harvested on day 16 post-immunisation (equivalent to the EAE peak stage). All tissues were homogenised to determine the levels of both pro-IL-16 and bioactive IL-16 by ELISA (Figure 1F). Spinal cord tissues of MOG_35-55_-immunised mice showed significantly elevated levels of IL-16 on day 12 and day 16 compared to day 16 PBS control mice. IL-16 levels were then substantially reduced on day 26 relative to day 12 and day 16. While IL-16 was detected in brain cerebellum and cerebrum tissues of PBS mice, the levels were significantly higher in these tissues of EAE day 12 and day 16 mice, decreasing towards control levels on day 26.

### 3.2. IL-16 and Its Receptor CD4 Expressing Cells Are Predominantly Located in the CNS Lesions

**Figure 2:**
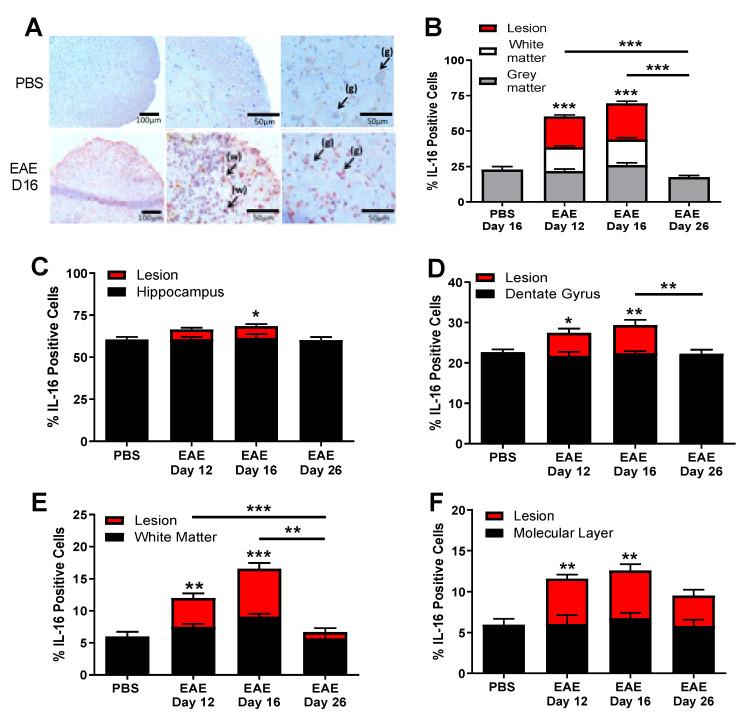
Expression of IL-16 in the CNS tissues of EAE mice. Spinal cord and brain tissues were harvested from MOG_35-55_ immunised mice on day 12, day 16 and day 28 or PBS immunised mice on day 16. Tissues were then stained with anti-IL-16 antibody and counterstained with haematoxylin to determine the expression of IL-16. (**A**) Representative images of spinal cord staining with IL-16 from tissues of different mice in EAE or PBS group groups; white matter (w) and grey matter (g) are indicated with arrows. (**B**–**F**) Percentage of IL-16 positive cells in the CNS tissues was qualified and results are expressed as the mean ± SEM. (**B**) Spinal cord; (**C**,**D**) Hippocampus; (**E**,**F**) cerebellum. N = 5 for all groups. Statistical significance was determined by one-way ANOVA with Bonferroni post hoc test. * *p* < 0.05, ** *p* < 0.01, *** *p* < 0.001 versus PBS or comparison groups are indicated by the bar lines.

Having shown significantly increased levels of IL-16 protein in the CNS tissues of EAE mice, we next investigated the location of IL-16 within the CNS in EAE and control mice, using immunohistochemical staining with an antibody which detects all forms of IL-16. In the spinal cord, the percentage of IL-16^+^ cells was comparable in the grey matter (GM) region between PBS and different stages of EAE mice (Figure 2A,B). However, there was a significant increase in the percentage of IL-16^+^ cells within the white matter (WM) and the lesion regions for EAE day 12 and day 16 mice compared to PBS controls as well as EAE day 26 mice, with both of these groups demonstrating few IL-16 expressing cells (Figure 2A,B).

IL-16 expression was also examined in sagittal brain sections, specifically the hippocampus and the cerebellum regions where the immune cell infiltration was particularly evident. In the hippocampus (Figure 2C,D), IL-16 was found to be highly expressed in the CA1-CA3 and dentate gyrus regions in all groups of mice. The percentage of IL-16^+^ cells within the hippocampus of EAE mice was significantly increased in clusters of infiltrating cells within lesions for day 12 and day 16 EAE mice (Figure 2C). In the dentate gyrus, cellular infiltration and lesions were observed with significantly increased percentage of IL-16^+^ cells in EAE day 12 and day 16 mice compared to PBS controls (Figure 2D), although in the day 26 EAE resolution stage, the percentage of IL-16^+^ cells was similar to control values. The IL-16 expression level in the hippocampus and dentate gyrus without lesions was comparable between all EAE groups and PBS controls.

In the cerebellum (Figure 2E,F), IL-16 was constitutively expressed in both control and EAE groups, with the percentage of IL-16^+^ cells significantly increased in tissues of day 12 and day 16 EAE mice in comparison to PBS control mice, with no difference observed between day 26 EAE and control mice. Analysis of IL-16^+^ cells in the WM (Figure 2E) and molecular layer (ML, Figure 2F) of the cerebellum revealed significant increases in the percentage of IL-16^+^ cells in EAE groups (days 12 and 16) compared to PBS controls, whereas EAE day 26 levels were not significantly changed relative to PBS mice. Furthermore, the significant increase correlated with the lesions in the WM and ML, as the IL-16 expression level in the cerebellum without lesions was comparable between all EAE groups and PBS controls.

**Figure 3:**
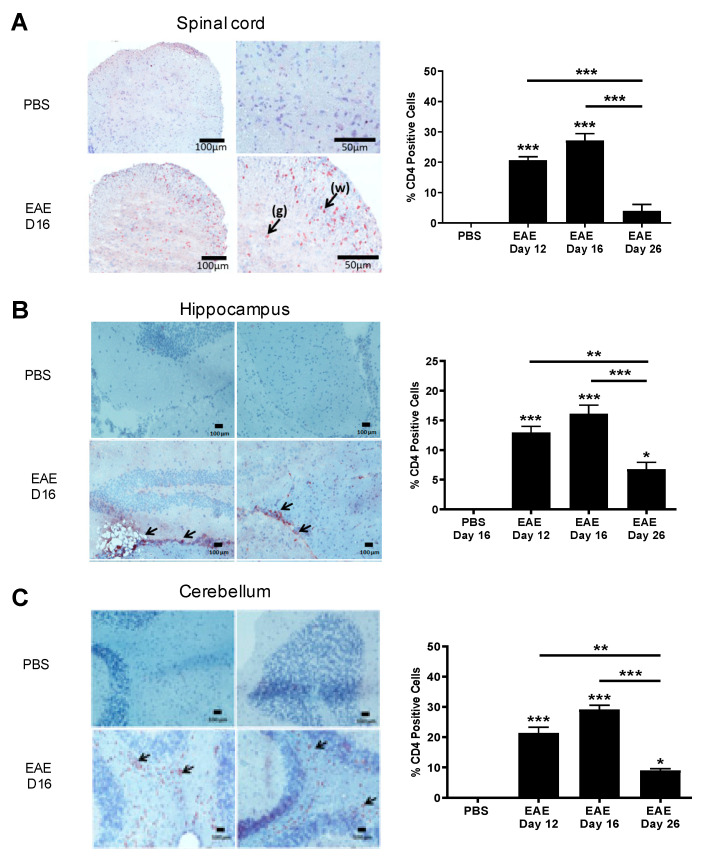
Expression of CD4 in the CNS tissues of EAE mice. Brain and spinal cord tissues harvested from EAE and PBS control mice were stained with anti-CD4 antibody and counterstained with haematoxylin in (**A**) spinal cord, (**B**) hippocampus or (**C**) cerebellum. Representative images are shown. Percentage of CD4 positive cells in each group was quantified with results expressed as the mean ± SEM. N = 5. Statistical significance was determined by one-way ANOVA with Bonferroni post hoc tests. * *p* < 0.05, ** *p* < 0.01, *** *p* < 0.001 versus PBS or comparison groups are indicated by the bar lines.

We next studied the expression of IL-16 receptor CD4 in CNS tissues of control and EAE mice. CD4^+^ cells were detected in EAE spinal cord tissue during all three time points but were absent in PBS controls (Figure 3A). However, a significantly higher percentage of CD4^+^ cells was observed within close proximity of the inflammatory WM lesions of EAE groups (day 12 and day 16) in comparison to the day 26 resolution stage of EAE mice with only small clusters of few cells shown within the WM of the spinal cord. Similar findings were observed in the hippocampus (Figure 3B) and cerebellum (Figure 3C) regions of EAE mice, with the majority of the CD4^+^ cells observed within close proximity of inflammatory lesions.

### 3.3. Increased IL-16 Expression Co-Localises with Infiltrating Immune Cells

**Figure 4:**
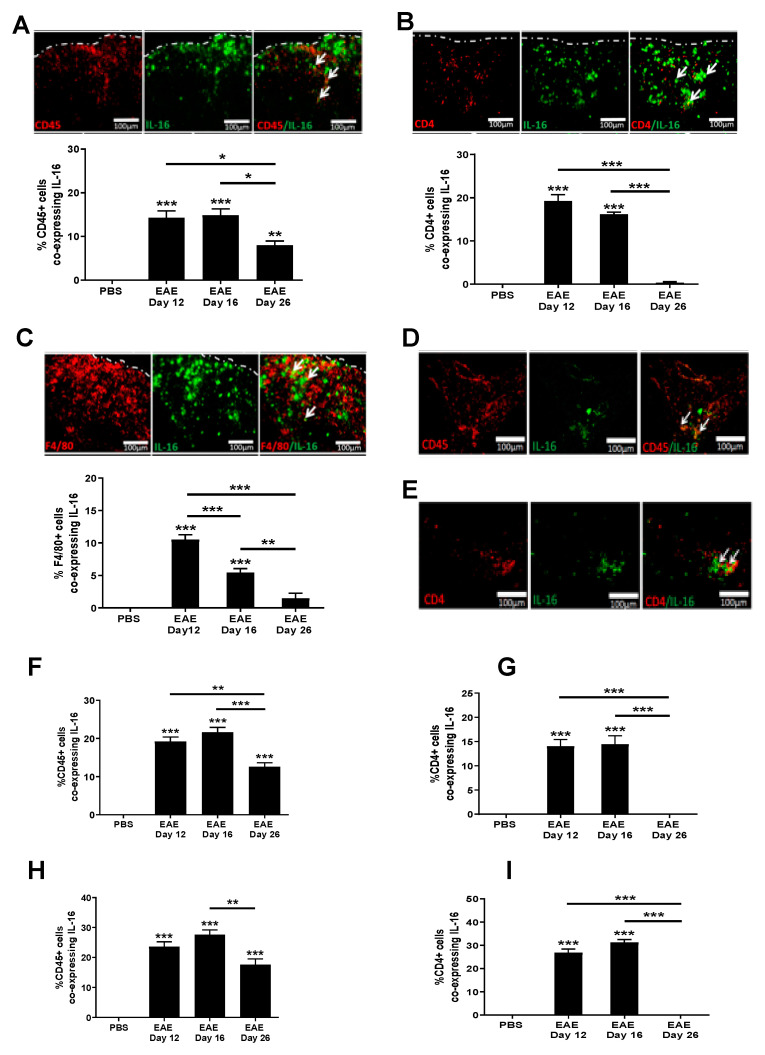
Expression of IL-16 by infiltrating immune cells in EAE mice. Spinal cord and brains were harvested from EAE and PBS control mice. Tissue was co-stained with IL-16 (green) and CD45, CD4, or F4/80 (all red). Representative images of spinal cord (**A**–**C**), cerebellum (**D**) and hippocampus (**E**) tissues are shown. Dotted white lines indicate spinal cord section edges. White arrows indicate areas of colocalization. Percentage of double-positive cells in each group within the spinal cord (**A**–**C**), hippocampus (**F**,**G**) and cerebellum (**H**,**I**) were quantified with results expressed as the mean ± SEM. N = 5. Statistical significance was determined by one-way ANOVA with Bonferroni post hoc tests. * *p* < 0.05, ** *p* < 0.01, *** *p* < 0.001 versus PBS or comparison groups are indicated by the bar lines.

Having established increased IL-16 expression in the CNS of EAE mice, we next sought to identify the cells that are associated with this increase. Our data show there was little colocalisation of IL-16 with CD45, CD4, F4/80 positive cells in the CNS of control mice. However, IL-16 was expressed by a significant percentage of CD45^+^ cells within the lesions predominantly in the WM of spinal cord tissues in all EAE group mice (Figure 4A). The percentage of IL-16^+^ CD45^+^ co-expressing cells was significantly higher in EAE day 12 and EAE day 16 groups, and in the EAE day 26 group, the percentage was reduced compared with day 12 and day 16. Further study using antibodies of CD4 and F4/80 suggests that these increased CD45^+^IL-16^+^ cells include those of CD4^+^ (Figure 4B) and F4/80^+^ (Figure 4C) infiltrating immune cells.

In the brain, IL-16 colocalised with CD45^+^ cells predominantly within lesions was observed in the dentate gyrus and throughout the cerebellar WM region of EAE mice (Figure 4D–I). Within hippocampal lesions (Figure 4F), IL-16^+^ CD45^+^ co-expressing cells was significantly elevated in EAE day 12 and day 16 tissues compared to the PBS control mice, which had few CD45^+^ IL-16^+^ cells. The percentage of IL-16^+^ CD45^+^ co-expressing cells was significantly reduced in tissues of day 26 EAE mice in comparison to that of day 12 and day 16 EAE mice, but was still significantly higher than the PBS control. Similar results were also observed in the cerebellum (Figure 4H).

Our data also show colocalisation of IL-16 with CD4^+^ cells predominantly within hippocampal (Figure 4G) and cerebellar (Figure 4I) lesion areas in EAE mice. Comparable levels of IL-16 expressing CD4^+^ cells were observed in EAE day 12 and day 16 mice, both significantly higher than that of the PBS control and day 26 EAE brain tissue. In contrast to EAE spinal cord sections, very few F4/80^+^ cells were detected in the brain tissues of EAE mice at all three time points; thus, no co-expression with IL-16 could be assessed.

### 3.4. Increased IL-16 Co-Localises with Glial Cells but Not Neurons

**Figure 5:**
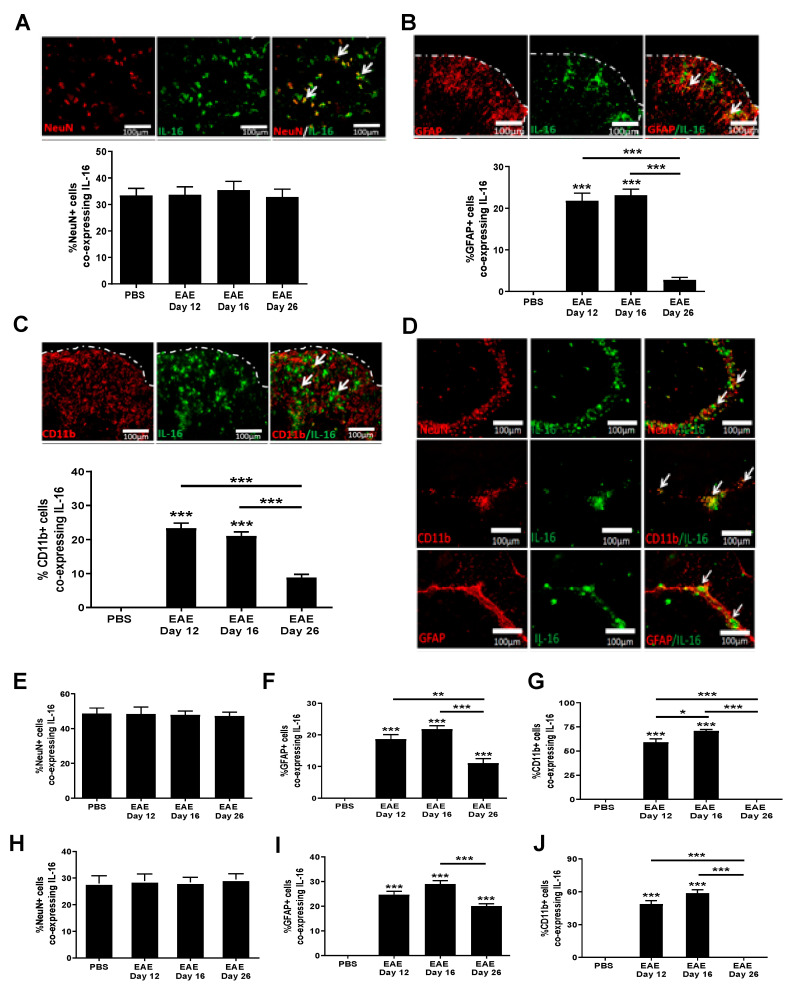
Expression of IL-16 by CNS resident cells in EAE mice. Spinal cord and brain tissues were harvested from EAE and PBS control mice and stained with IL-16 (green) together with NeuN, GFAP or CD11b (all red). Representative images of spinal cord (**A**–**C**) and hippocampus (**D**) tissues are shown. Dotted white lines indicate tissue section edges. White arrows indicate areas of colocalisation. Percentage of double-positive cells in each group within either the spinal cord (**A**–**C**) or hippocampus (**E**–**G**) and cerebellum (**H**–**J**) was quantified with results expressed as the mean ± SEM. N = 5. Statistical significance was determined by one-way ANOVA with Bonferroni post hoc tests. * *p* < 0.05, ** *p* < 0.01, *** *p* < 0.001 versus PBS or comparison groups are indicated by the bar lines.

Although IL-16 was clearly expressed by infiltrating immune cells within the CNS tissues of EAE mice, constitutive expression of IL-16 in the spinal cord and brain tissues across all groups suggests it is also expressed by CNS resident cells. Thus, next we studied the co-localization of IL-16 with neurons and glia in the CNS under normal and diseased conditions. Our data show that IL-16 co-localised with NeuN^+^ cells within the GM of spinal cord of all mice examined (Figure 5A), with the percentage of NeuN^+^ cells expressing IL-16 being comparable between all three different stages of EAE mice and the control group. However, compared to controls, the percentage of IL-16 expressing GFAP^+^ (Figure 5B) or CD11b^+^ (Figure 5C) cells was significantly increased in the spinal cords of day 12 and day 16 EAE mice, predominantly within the WM lesions, whereas on day 26, colocalisation had reduced to levels similar to those of control mice with few cells co-expressing both IL-16 and GFAP or IL-16 and CD11b.

The expression of IL-16 by CNS resident cells in the hippocampus and cerebellum mirrored that observed in the spinal cord, with both hippocampal and cerebellar sections containing a high percentage of NeuN^+^ neurons expressing IL-16 (Figure 5D,E,H). Comparable levels were observed between all groups—PBS control and various disease stages of EAE mice in both hippocampus and cerebellum regions. However the percentage of IL-16 expressing GFAP^+^ (Figure 5D,F,I) or CD11b^+^ (Figure 5D,G,J) cells was significantly increased in tissues of EAE mice at all three time points, with the highest percentage observed on day 16, and slightly reduced levels on day 12 and on day 26 in tissues of both the hippocampus and cerebellum. Few GFAP^+^ IL-16^+^ and CD11b^+^IL-16^+^ cells were observed in the tissues of PBS control mice.

## 4. Discussion

EAE is the most commonly used animal model for studying MS because of its close immunopathological resemblance to MS [2,23]. Here, we investigated the involvement of IL-16 in the development of neuroinflammation by studying the expression and cellular source of IL-16 within the CNS of MOG_35-55_ induced EAE mice.

While the roles of many immune cytokines such as IFN-γ, IL-17 and IL-10 in the pathogenesis of MS/EAE are well documented [22,24], information is less clear for IL-16. Several studies suggest a possible role of IL-16 in MS/EAE development [16,25], so understanding of its function may provide new insights into the immunopathogenesis of MS. IL-16 is produced by many cells including monocytes, mast cells and fibroblasts and is a cytokine constitutive to T lymphocytes [12,25]. IL-16 has pleiotropic roles in the biological functions of CD4^+^ T cells and therefore likely plays an important role in immune-mediated diseases [8,12]. Studies using western blot show that pro-IL-16 and bioactive IL-16 are expressed in the spleen and lymph node tissues of both relapsing and chronic EAE mice, surprisingly with little difference in the expression levels between them [16], indicating the expression levels of IL-16 in the lymphoid organs are likely not affected by the activation status of the immune cells. The findings are supported by our ELISA data of the similar levels of overall IL-16 production by the splenocytes of PBS and MOG_35-55_ immunised mice and between cultures treated with medium, MOG_35-55_ antigen or ConA. However, T cells isolated from the spleen and inguinal lymph nodes of naive and MOG_35-55_ immunised mice have been shown to produce elevated levels of IL-16 upon in-vitro re-stimulation with PHA (naïve mice cells) and MOG_35-55_ antigen or PHA (EAE mice cells) in comparison to naïve control T cells [16]. These data suggest while activated T cells express higher levels of IL-16 in response to antigen specific or non-specific stimulation, the change may not be sufficient to influence the overall level of IL-16 expressed/produced by the lymphoid organs which include different types of cells, many of which are important sources of IL-16 production [26,27]. The findings may also suggest a functional difference between IL-16 and the other well-studied immune cytokines such as IFN-γ and IL-17 [22] during the development of inflammatory diseases such as MS/EAE.

Skundric et al. [16] reported in 2005 that a significant upregulation of bioactive IL-16 was observed in the spinal cord tissues of acute, relapsing and chronic EAE mice in comparison to controls using western blot analysis, with the highest observed in the relapsing mice. Their findings were confirmed in MS patients using the same method, with increased levels of both pro-IL-16 and bioactive IL-16 observed in the CNS lesions of patients compared to controls, with the levels peaked in acute and diminished in subacute lesions [12,25]. Our findings from examining the protein levels of IL-16 in the supernatants of the spinal cord and brain cerebellum and cerebrum homogenates using ELISA agree with these reports that the levels of IL-16 protein in the CNS were significantly increased in the EAE mice at onset and peak stages in comparison to PBS immunised control mice, with the highest levels in the tissues of EAE peak mice. These findings suggest a close correlation of IL-16 expression levels in the CNS with the activity of neuroinflammation.

The upregulation of IL-16 in the CNS tissues of EAE mice was further confirmed by immunohistochemical staining which revealed a significantly higher percentage of IL-16^+^ cells within the spinal cord of EAE mice particularly at the disease initiation and peak time points, in comparison to PBS controls. Further analysis suggests that the increased percentage of IL-16^+^ cells was primarily found within the inflammatory lesion in comparison to the equivalent area in the spinal cord, indicating a direct link between IL-16^+^ cells and neuroinflammation. Indeed, very few cells were IL-16^+^ in the tissues of PBS controls and EAE resolution stage mice that had no or few lesions. Consistent findings of increased percentages of IL-16^+^ cells within the inflammatory lesions were also observed within the cerebellum and the hippocampus, the brain regions closely associated with MS disease [28,29,30], of MOG_35-55_ immunised EAE mice.

The predominant expression of IL-16 in the lesions of CNS WM regions of EAE mice suggests that IL-16 is likely to be expressed by the infiltrating immune cells within these areas; this was confirmed by the significantly increased percentage of CD45^+^ cells co-expressing IL-16. Furthermore, the immunohistochemical staining data suggest that IL-16 is likely expressed by various immune cells including CD4^+^ cells and F4/80^+^ macrophages, as well as CD11b^+^ monocytes/microglia cells, and GFAP^+^ astrocytes in the CNS. The colocalisation of IL-16 and CD4 in the brain and spinal cord tissues of EAE mice is not surprising as IL-16 was co-immunoprecipitated with CD4 in the CNS of EAE mice previously [16] and is a CD4^+^ T cell-specific chemokine attractant cytokine [18,31]. While the IL-16 antibody we used was not able to distinguish between pro-IL-16 and bioactive IL-16, Skundric et al. suggested that bioactive IL-16 was produced locally in the CNS by infiltrating CD4^+^ T cells, as they reported the colocalisation of IL-16 with active caspase-3 in the mononuclear cells of EAE spinal cord [16]. This could explain the correlation of IL-16 expression levels in the CNS with the severity of neuroinflammation indicated by the number of infiltrating CD4^+^ cells shown in this study and previously [21]. However, the upregulation of IL-16 was not limited to CD4^+^ cells in the CNS lesion of EAE mice; IL-16 is reported to be expressed by B220^+^ B cells, CD8^+^ T cells and granulocytes [18,32]. Our study also reveals other cellular sources of IL-16 from monocyte/macrophages, as upregulated expression of IL-16 was observed in the infiltrating monocytes and CNS resident microglia and astrocytes during the neuroinflammation of EAE mice. Our findings agree with previous reports indicating the expression of IL-16 by activated CD68^+^ microglia/macrophages in the brain of focal cerebral infarction patients [32] or by the *in vitro* cultured microglia and macrophage cells [33]. The data may suggest a key role IL-16 plays during the development of neuroinflammation through the recruitment and activation of CD4^+^ T cells possibly via both autocrine and paracrine manners.

The presence of IL-16 in the CNS tissues of PBS control mice while its receptor CD4 was absent indicates an alternate function of this cytokine in the CNS under a normal physiological state. IL-16 is shown to be expressed in the CA1, CA2 and CA3 regions of hippocampus, as well as the dentate gyrus. Within the cerebellum region, IL-16 was dispersed throughout the molecular layer and in the granule cells within the granular layer. Indeed, the expression of IL-16 is confirmed to be with NeuN+ neurons in both the spinal cord and the brain. However, the expression level and pattern of IL-16 in neurons remain the same throughout the EAE course. Thus, alterations in IL-16 expression in the CNS during EAE are likely to originate from the infiltrating immune cells and the activated glial cells, as the upregulation was predominantly found in the WM of the CNS tissues where the cellular infiltration and the lesions were observed. The exact effect of IL-16 on neurons is not known; we previously reported that IL-16 reduces hippocampal neuronal excitatory and synaptic activity via CD4- and CD9-independent inhibition of sodium channel function and GluA1 phosphorylation [20]. Therefore, further study is important to look at the CNS-specific function of IL-16 under normal and neuroinflammatory conditions, which will also provide new insights into the role of IL-16 in MS disease.

In conclusion, this study demonstrates a close correlation between the elevated expression of IL-16 in the CNS by the infiltrating immune cells such as CD4^+^ cells, macrophages and the CNS resident microglia and astrocytes, with increased neuroinflammation in EAE. Our findings suggest that the IL-16/CD4 signalling pathway is closely involved in the neuroinflammation of MS and other neurological disorders through the immune and CNS resident cells.
